# The genome sequence of the Broom moth, *Ceramica pisi *Linnaeus, 1758

**DOI:** 10.12688/wellcomeopenres.23050.1

**Published:** 2024-09-20

**Authors:** Andy Griffiths, Denise C. Wawman, Liam M. Crowley, University of Oxford and Wytham Woods Genome Acquisition Lab, Wellcome Sanger Institute Tree of Life Management, Samples and Laboratory team, Wellcome Sanger Institute Scientific Operations: Sequencing Operations, Wellcome Sanger Institute Tree of Life Core Informatics team, Tree of Life Core Informatics collective, Darwin Tree of Life Consortium

**Affiliations:** 1 Wellcome Sanger Institute, Hinxton, England, UK; 2 Royal Botanic Garden Edinburgh, Edinburgh, Scotland, UK; 3 Department of Biology, University of Oxford, Oxford, England, UK

**Keywords:** Ceramica pisi, Broom moth, genome sequence, chromosomal, Lepidoptera

## Abstract

We present a genome assembly from an individual male *Ceramica pisi* (the Broom moth; Arthropoda; Insecta; Lepidoptera; Noctuidae). The genome sequence spans 732.70 megabases. Most of the assembly is scaffolded into 31 chromosomal pseudomolecules, including the Z sex chromosome. The mitochondrial genome has also been assembled and is 15.31 kilobases in length. Gene annotation of this assembly on Ensembl identified 12,916 protein-coding genes.

## Species taxonomy

Eukaryota; Opisthokonta; Metazoa; Eumetazoa; Bilateria; Protostomia; Ecdysozoa; Panarthropoda; Arthropoda; Mandibulata; Pancrustacea; Hexapoda; Insecta; Dicondylia; Pterygota; Neoptera; Endopterygota; Amphiesmenoptera; Lepidoptera; Glossata; Neolepidoptera; Heteroneura; Ditrysia; Obtectomera; Noctuoidea; Noctuidae; Hadeninae; *Ceramica*; *Ceramica pisi* Linnaeus, 1758 (NCBI:txid988087).

## Background

**Figure 1.**
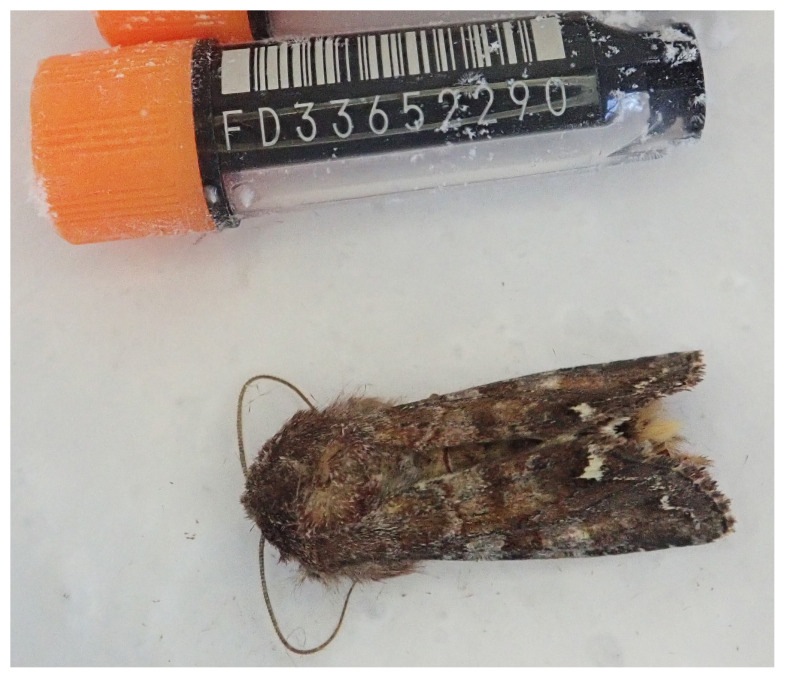
Photograph of the *Ceramica pisi* (ilCerPisi3) specimen used for RNA sequencing.

The genome of the Broom moth, *Ceramica pisi* ( Figure 1), was sequenced as part of the Darwin Tree of Life Project ( Blaxter *et al.*, 2022), a collaborative effort to sequence all named eukaryotic species in the Atlantic Archipelago of Britain and Ireland. Here we present a chromosomally complete genome sequence for *Ceramica pisi*, based on one male specimen from Carrifran Wildwood, Scotland, UK.

## Genome sequence report

The genome of an adult male *Ceramica pisi* was sequenced using Pacific Biosciences single-molecule HiFi long reads, generating a total of 76.09 Gb (gigabases) from 7.62 million reads, providing approximately 117-fold coverage. Primary assembly contigs were scaffolded with chromosome conformation Hi-C data, which produced 99.09 Gb from 656.19 million reads, yielding an approximate coverage of 135-fold. Specimen and sequencing information is summarised in Table 1.

**Table 1.** Specimen and sequencing data for *Ceramica pisi*.

Project information			
**Study title**	Ceramica pisi (broom moth)		
**Umbrella BioProject**	PRJEB65408		
**Species**	*Ceramica pisi*		
**BioSample**	SAMEA112198383		
**NCBI taxonomy ID**	988087		
Specimen information			
**Technology**	**ToLID**	**BioSample accession**	**Organism part**
**PacBio long read sequencing**	ilCerPisi1	SAMEA112198456	thorax
**Hi-C sequencing**	ilCerPisi2	SAMEA112198479	head
**RNA sequencing**	ilCerPisi3	SAMEA112232914	abdomen
Sequencing information			
**Platform**	**Run accession**	**Read count**	**Base count (Gb)**
**Hi-C Illumina NovaSeq 6000**	ERR11904130	6.56e+08	99.09
**PacBio Sequel IIe**	ERR11892488	1.57e+06	14.34
**PacBio Revio**	ERR11892487	7.62e+06	76.09
**RNA Illumina NovaSeq 6000**	ERR12035204	5.78e+07	8.73

**Figure 2.**
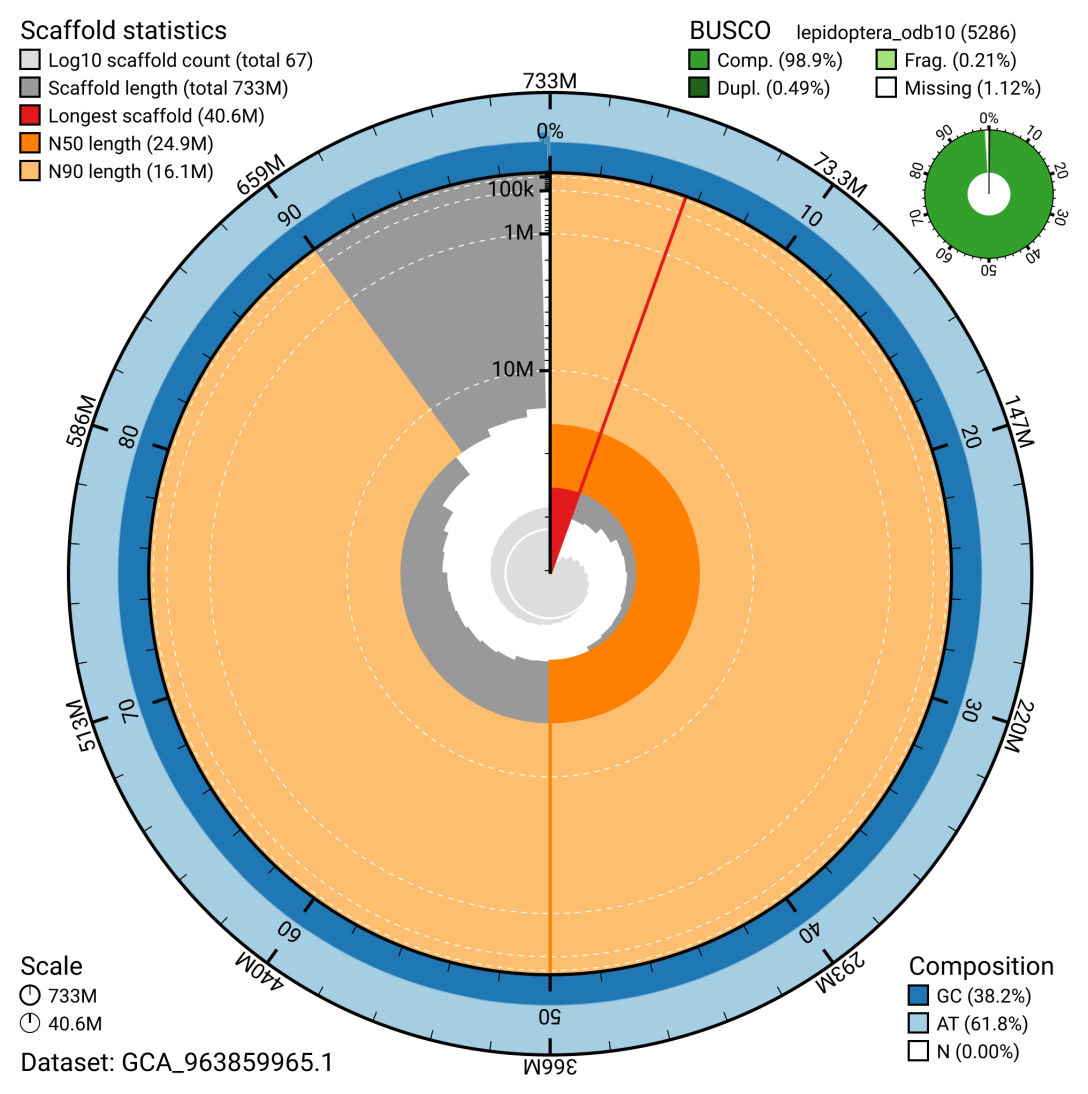
Genome assembly of *Ceramica pisi*, ilCerPisi1.1: metrics. The BlobToolKit snail plot shows N50 metrics and BUSCO gene completeness. The main plot is divided into 1,000 size-ordered bins around the circumference with each bin representing 0.1% of the 732,715,256 bp assembly. The distribution of scaffold lengths is shown in dark grey with the plot radius scaled to the longest scaffold present in the assembly (40,616,997 bp, shown in red). Orange and pale-orange arcs show the N50 and N90 scaffold lengths (24,874,717 and 16,061,681 bp), respectively. The pale grey spiral shows the cumulative scaffold count on a log scale with white scale lines showing successive orders of magnitude. The blue and pale-blue area around the outside of the plot shows the distribution of GC, AT and N percentages in the same bins as the inner plot. A summary of complete, fragmented, duplicated and missing BUSCO genes in the lepidoptera_odb10 set is shown in the top right. An interactive version of this figure is available at https://blobtoolkit.genomehubs.org/view/GCA_963859965.1/dataset/GCA_963859965.1/snail.

**Figure 3.**
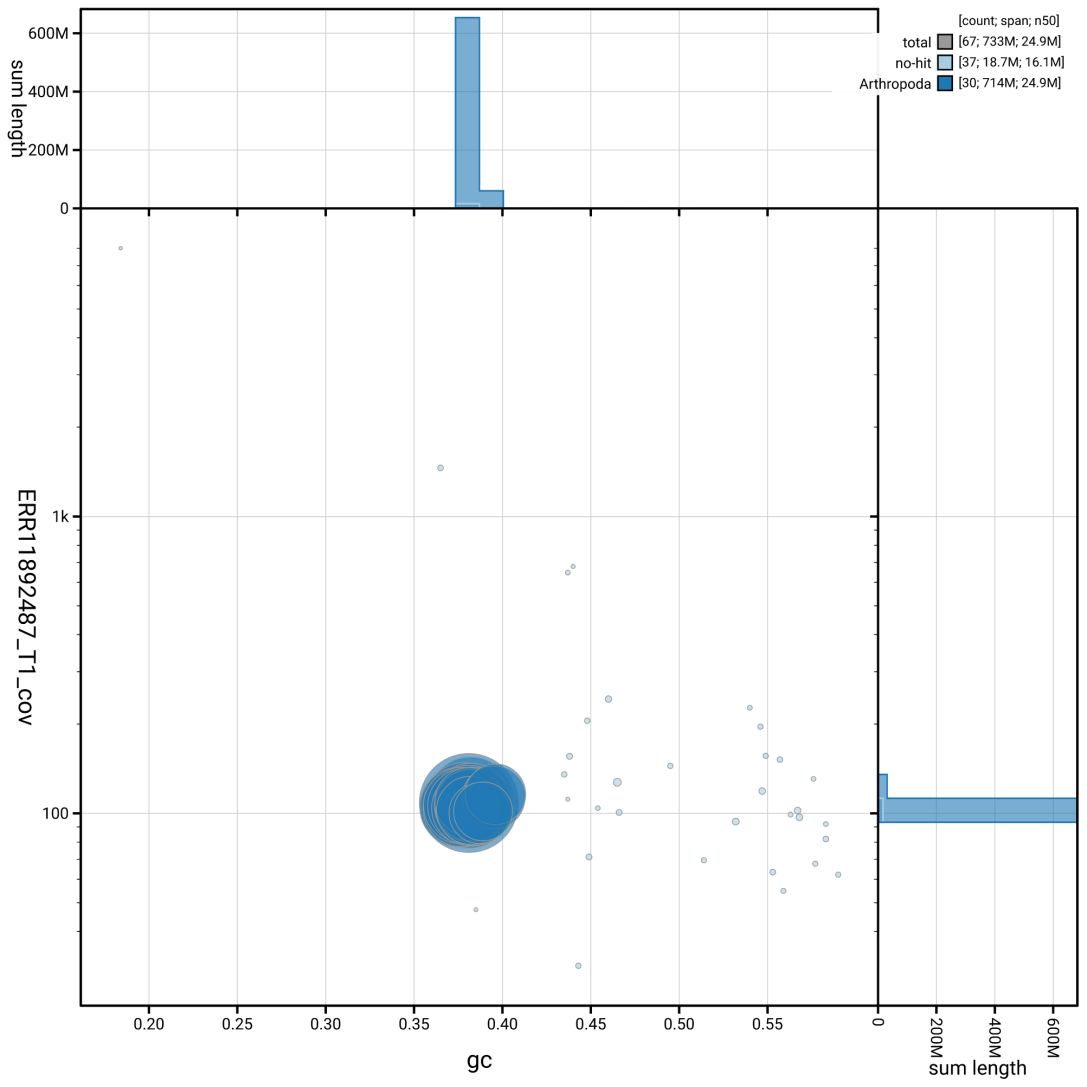
Genome assembly of *Ceramica pisi*, ilCerPisi1.1: BlobToolKit GC-coverage plot. Base coverage is plotted on the vertical axis, while the horizontal axis shows GC%. Sequences are coloured by phylum. Circles are sized in proportion to sequence length. Histograms show the distribution of sequence length sum along each axis. An interactive version of this figure is available at https://blobtoolkit.genomehubs.org/view/GCA_963859965.1/dataset/GCA_963859965.1/blob.

**Figure 4.**
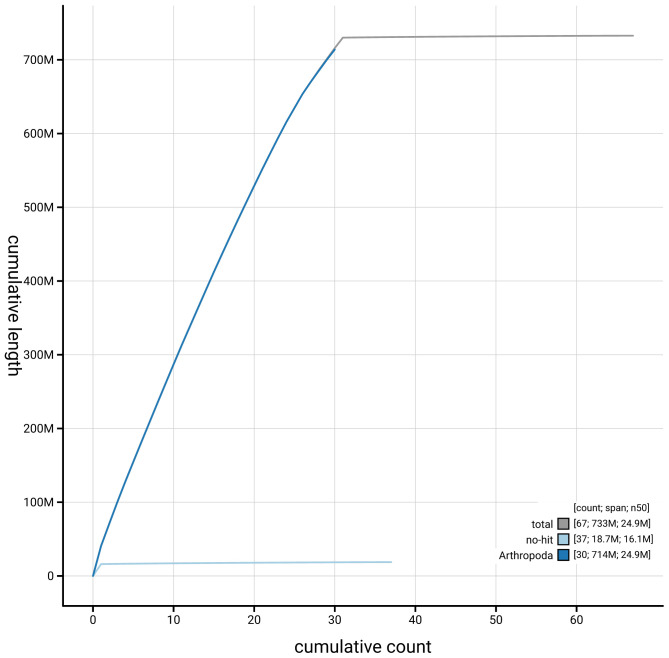
Genome assembly of *Ceramica pisi* ilCerPisi1.1: BlobToolKit cumulative sequence plot. The grey line shows cumulative length for all sequences. Coloured lines show cumulative lengths of sequences assigned to each phylum using the buscogenes taxrule. An interactive version of this figure is available at https://blobtoolkit.genomehubs.org/view/GCA_963859965.1/dataset/GCA_963859965.1/cumulative.

**Figure 5.**
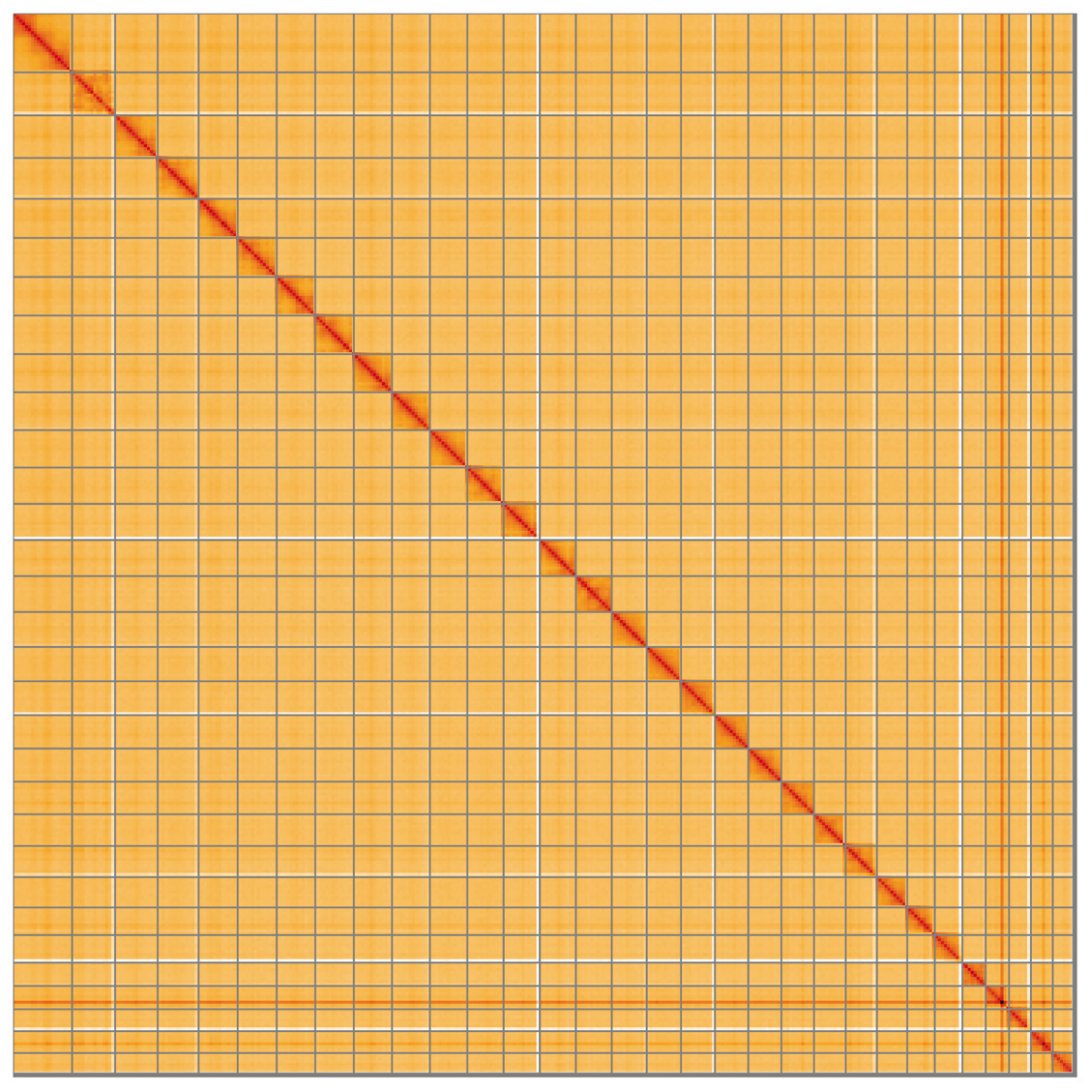
Genome assembly of *Ceramica pisi* ilCerPisi1.1: Hi-C contact map of the ilCerPisi1.1 assembly, visualised using HiGlass. Chromosomes are shown in order of size from left to right and top to bottom. An interactive version of this figure may be viewed at https://genome-note-higlass.tol.sanger.ac.uk/l/?d=N0lSy7fGQ7SSE1afN54MCg.

Manual assembly curation corrected 25 missing joins or mis-joins and 7 haplotypic duplications, reducing the assembly length by 0.65% and the scaffold number by 1.47%. The final assembly has a total length of 732.70 Mb in 66 sequence scaffolds, with 46 gaps, and a scaffold N50 of 24.9 Mb ( Table 2). The snail plot in Figure 2 provides a summary of the assembly statistics, while the distribution of assembly scaffolds on GC proportion and coverage is shown in Figure 3. The cumulative assembly plot in Figure 4 shows curves for subsets of scaffolds assigned to different phyla. Most (99.63%) of the assembly sequence was assigned to 31 chromosomal-level scaffolds, representing 30 autosomes and the Z sex chromosome. Chromosome-scale scaffolds confirmed by the Hi-C data are named in order of size ( Figure 5; Table 3). The Z chromosome was identified based on synteny with *Orthosia gothica* (GCA_949775005.1) ( Boyes *et al.*, 2024). While not fully phased, the assembly deposited is of one haplotype. Contigs corresponding to the second haplotype have also been deposited. The mitochondrial genome was also assembled and can be found as a contig within the multifasta file of the genome submission.

**Table 2.** Genome assembly data for *Ceramica pisi*, ilCerPisi1.1.

Genome assembly		
Assembly name	ilCerPisi1.1	
Assembly accession	GCA_963859965.1	
*Accession of alternate haplotype*	*GCA_963859975.1*	
Span (Mb)	732.70	
Number of contigs	113	
Contig N50 length (Mb)	13.5	
Number of scaffolds	66	
Scaffold N50 length (Mb)	24.9	
Longest scaffold (Mb)	40.62	
Assembly metrics *		*Benchmark*
Consensus quality (QV)	68.5	*≥ 50*
*k*-mer completeness	100.0%	*≥ 95%*
BUSCO **	C:98.9%[S:98.4%,D:0.5%], F:0.2%,M:0.9%,n:5,286	*C ≥ 95%*
Percentage of assembly mapped to chromosomes	99.63%	*≥ 95%*
Sex chromosomes	Z	*localised homologous pairs*
Organelles	Mitochondrial genome: 15.31 kb	*complete single alleles*
Genome annotation of assembly GCA_963859965.1 at Ensembl		
Number of protein-coding genes	12,916	
Number of non-coding genes	2,072	
Number of gene transcripts	23,379	

* Assembly metric benchmarks are adapted from column VGP-2020 of “Table 1: Proposed standards and metrics for defining genome assembly quality” from Rhie *et al.* (2021). ** BUSCO scores based on the lepidoptera_odb10 BUSCO set using version 5.4.3. C = complete [S = single copy, D = duplicated], F = fragmented, M = missing, n = number of orthologues in comparison. A full set of BUSCO scores is available at https://blobtoolkit.genomehubs.org/view/Ceramica_pisi/dataset/GCA_963859965.1/busco.

**Table 3.** Chromosomal pseudomolecules in the genome assembly of *Ceramica pisi*, ilCerPisi1.

INSDC accession	Name	Length (Mb)	GC%
OY982528.1	1	29.67	38.0
OY982529.1	2	29.29	38.0
OY982530.1	3	28.08	38.0
OY982531.1	4	27.0	38.5
OY982532.1	5	26.83	37.5
OY982533.1	6	26.61	38.0
OY982534.1	7	26.52	37.5
OY982535.1	8	26.25	38.0
OY982536.1	9	26.11	38.0
OY982537.1	10	25.82	38.0
OY982538.1	11	24.99	38.0
OY982539.1	12	24.89	38.0
OY982540.1	13	24.87	38.0
OY982541.1	14	24.74	37.5
OY982542.1	15	24.02	37.5
OY982543.1	16	23.69	38.0
OY982544.1	17	23.33	38.0
OY982545.1	18	22.96	38.5
OY982546.1	19	22.74	38.0
OY982547.1	20	22.54	38.5
OY982548.1	21	21.82	38.5
OY982549.1	22	21.54	38.0
OY982550.1	23	20.79	38.0
OY982551.1	24	19.0	38.0
OY982552.1	25	18.97	38.0
OY982553.1	26	16.06	38.5
OY982554.1	27	16.06	39.5
OY982555.1	28	15.12	38.5
OY982556.1	29	15.0	39.5
OY982557.1	30	14.1	39.0
OY982527.1	Z	40.62	38.0
OY982558.1	MT	0.02	18.5

The estimated Quality Value (QV) of the final assembly is 68.5 with *k*-mer completeness of 100.0%, and the assembly has a BUSCO v5.4.3 completeness of 98.9% (single = 98.4%, duplicated = 0.5%), using the lepidoptera_odb10 reference set ( *n* = 5,286).

Metadata for specimens, BOLD barcode results, spectra estimates, sequencing runs, contaminants and pre-curation assembly statistics are given at https://links.tol.sanger.ac.uk/species/988087.

## Genome annotation report

The *Ceramica pisi* genome assembly (GCA_963859965.1) was annotated at the European Bioinformatics Institute (EBI) on Ensembl Rapid Release. The resulting annotation includes 23,379 transcribed mRNAs from 12,916 protein-coding and 2,072 non-coding genes ( Table 2; https://rapid.ensembl.org/Ceramica_pisi_GCA_963859965.1/Info/Index). The average transcript length is 19,603.74. There are 1.56 coding transcripts per gene and 7.28 exons per transcript.

## Methods

### Sample acquisition

Specimens of *Ceramica pisi* were collected from Carrifran Wildwood, Moffat Hills, Scotland, UK (latitude 55.41, longitude –3.34) on 2022-06-23 using a moth trap. The specimen was collected and identified by Andy Griffiths (Wellcome Sanger Institute) and preserved by flash freezing. Specimen ID SAN00002576 (ToLID ilCerPisi1) was used for PacBio HiFi sequencing and specimen ID SAN00002580 (ToLID ilCerPisi2).

The specimen used for RNA sequencing (specimen ID Ox002238, ToLID ilCerPisi3) was an adult specimen collected from Bratton, Somerset, UK (latitude 51.16, longitude –4.67) on 2022-06-20 by light trap. the specimen was collected by Denise Wawman (University of Oxford) and identified by Liam Crowley (University of Oxford) and preserved on dry ice.

### Nucleic acid extraction

The workflow for high molecular weight (HMW) DNA extraction at the Wellcome Sanger Institute (WSI) Tree of Life Core Laboratory includes a sequence of core procedures: sample preparation and homogenisation, DNA extraction, fragmentation and purification. Detailed protocols are available on protocols.io ( Denton *et al.*, 2023b). The ilCerPisi1 sample was weighed and dissected on dry ice ( Jay *et al.*, 2023). Tissue from the thorax was homogenised using a PowerMasher II tissue disruptor ( Denton *et al.*, 2023a).

HMW DNA was extracted in the WSI Scientific Operations core using the Automated MagAttract v2 protocol ( Oatley *et al.*, 2023). The DNA was sheared into an average fragment size of 12–20 kb in a Megaruptor 3 system ( Bates *et al.*, 2023). Sheared DNA was purified by solid-phase reversible immobilisation, using AMPure PB beads to eliminate shorter fragments and concentrate the DNA ( Strickland *et al.*, 2023). The concentration of the sheared and purified DNA was assessed using a Nanodrop spectrophotometer and Qubit Fluorometer using the Qubit dsDNA High Sensitivity Assay kit. Fragment size distribution was evaluated by running the sample on the FemtoPulse system.

RNA was extracted from abdomen tissue of ilCerPisi3 ( Figure 1) in the Tree of Life Laboratory at the WSI using the RNA Extraction: Automated MagMax™ *mir*Vana protocol ( do Amaral *et al.*, 2023). The RNA concentration was assessed using a Nanodrop spectrophotometer and a Qubit Fluorometer using the Qubit RNA Broad-Range Assay kit. Analysis of the integrity of the RNA was done using the Agilent RNA 6000 Pico Kit and Eukaryotic Total RNA assay.

### Sequencing

Pacific Biosciences HiFi circular consensus DNA sequencing libraries were constructed according to the manufacturers’ instructions. Poly(A) RNA-Seq libraries were constructed using the NEB Ultra II RNA Library Prep kit. DNA and RNA sequencing was performed by the Scientific Operations core at the WSI on Pacific Biosciences Revio (HiFi) and Illumina NovaSeq 6000 (RNA-Seq) instruments. 

Hi-C data were generated from frozen head tissue of head tissue of ilCerPisi2 using the Arima-HiC v2 kit. In brief, frozen tissue (–80 °C) was fixed, and the DNA crosslinked using a TC buffer containing formaldehyde. The crosslinked DNA was then digested using a restriction enzyme master mix. The 5’-overhangs were then filled in and labelled with a biotinylated nucleotide and proximally ligated. The biotinylated DNA construct was fragmented to a fragment size of 400 to 600 bp using a Covaris E220 sonicator. The DNA was then enriched, barcoded, and amplified using the NEBNext Ultra II DNA Library Prep Kit, following manufacturers’ instructions. The Hi-C sequencing was performed using paired-end sequencing with a read length of 150 bp on an Illumina NovaSeq 6000 instrument.

### Genome assembly, curation and evaluation

***Assembly***

The HiFi reads were first assembled using Hifiasm ( Cheng *et al.*, 2021) with the --primary option. Haplotypic duplications were identified and removed using purge_dups ( Guan *et al.*, 2020). The Hi-C reads were mapped to the primary contigs using bwa-mem2 ( Vasimuddin *et al.*, 2019). The contigs were further scaffolded using the provided Hi-C data ( Rao *et al.*, 2014) in YaHS ( Zhou *et al.*, 2023) using the --break option. The scaffolded assemblies were evaluated using Gfastats ( Formenti *et al.*, 2022), BUSCO ( Manni *et al.*, 2021) and MERQURY.FK ( Rhie *et al.*, 2020).

The mitochondrial genome was assembled using MitoHiFi ( Uliano-Silva *et al.*, 2023), which runs MitoFinder ( Allio *et al.*, 2020) and uses these annotations to select the final mitochondrial contig and to ensure the general quality of the sequence.

***Assembly curation***

The assembly was decontaminated using the Assembly Screen for Cobionts and Contaminants (ASCC) pipeline (article in preparation). Flat files and maps used in curation were generated in TreeVal ( Pointon *et al.*, 2023). Manual curation was primarily conducted using PretextView ( Harry, 2022), with additional insights provided by JBrowse2 ( Diesh *et al.*, 2023) and HiGlass ( Kerpedjiev *et al.*, 2018). Scaffolds were visually inspected and corrected as described by Howe *et al*. (2021). Any identified contamination, missed joins, and mis-joins were corrected, and duplicate sequences were tagged and removed. The Z chromosome was identified based on synteny analysis. The curation process is documented at https://gitlab.com/wtsi-grit/rapid-curation (article in preparation).

***Evaluation of the final assembly***

The final assembly was post-processed and evaluated using the three Nextflow ( Di Tommaso *et al.*, 2017) DSL2 pipelines: sanger-tol/readmapping ( Surana *et al.,* 2023a), sanger-tol/genomenote ( Surana *et al.,* 2023b), and sanger-tol/blobtoolkit ( Muffato *et al.*, 2024). The readmapping pipeline aligns the Hi-C reads using bwa-mem2 ( Vasimuddin *et al.*, 2019) and combines the alignment files with SAMtools ( Danecek *et al.*, 2021). The genomenote pipeline transforms the Hi-C alignments into a contact map with BEDTools ( Quinlan & Hall, 2010) and the Cooler tool suite ( Abdennur & Mirny, 2020). The contact map is visualised in HiGlass ( Kerpedjiev *et al.*, 2018). This pipeline also generates assembly statistics using the NCBI datasets report ( Sayers *et al.*, 2024), computes *k*-mer completeness and QV consensus quality values with FastK and MERQURY.FK, and runs BUSCO ( Manni *et al.*, 2021) to assess completeness.

The sanger-tol/blobtoolkit pipeline is a Nextflow port of the previous Snakemake Blobtoolkit pipeline ( Challis *et al.*, 2020). It aligns the PacBio reads in SAMtools and minimap2 ( Li, 2018) and generates coverage tracks for regions of fixed size. In parallel, it queries the GoaT database ( Challis *et al.*, 2023) to identify all matching BUSCO lineages to run BUSCO ( Manni *et al.*, 2021). For the three domain-level BUSCO lineages, the pipeline aligns the BUSCO genes to the UniProt Reference Proteomes database ( Bateman *et al.*, 2023) with DIAMOND ( Buchfink *et al.*, 2021) blastp. The genome is also split into chunks according to the density of the BUSCO genes from the closest taxonomically lineage, and each chunk is aligned to the UniProt Reference Proteomes database with DIAMOND blastx. Genome sequences without a hit are chunked with seqtk and aligned to the NT database with blastn ( Altschul *et al.*, 1990). The blobtools suite combines all these outputs into a blobdir for visualisation.

The genome assembly and evaluation pipelines were developed using nf-core tooling ( Ewels *et al.*, 2020) and MultiQC ( Ewels *et al.*, 2016), relying on the Conda package manager, the Bioconda initiative ( Grüning *et al.*, 2018), the Biocontainers infrastructure ( da Veiga Leprevost *et al.*, 2017), as well as the Docker ( Merkel, 2014) and Singularity ( Kurtzer *et al.*, 2017) containerisation solutions.

Table 4 contains a list of relevant software tool versions and sources.

**Table 4.** Software tools: versions and sources.

Software tool	Version	Source
BEDTools	2.30.0	https://github.com/arq5x/bedtools2
BLAST	2.14.0	ftp://ftp.ncbi.nlm.nih.gov/blast/executables/blast+/
BlobToolKit	4.3.7	https://github.com/blobtoolkit/blobtoolkit
BUSCO	5.4.3 and 5.5.0	https://gitlab.com/ezlab/busco
bwa-mem2	2.2.1	https://github.com/bwa-mem2/bwa-mem2
Cooler	0.8.11	https://github.com/open2c/cooler
DIAMOND	2.1.8	https://github.com/bbuchfink/diamond
fasta_windows	0.2.4	https://github.com/tolkit/fasta_windows
FastK	427104ea91c78c3b8b8b49f1a7d6bbeaa869ba1c	https://github.com/thegenemyers/FASTK
Gfastats	1.3.6	https://github.com/vgl-hub/gfastats
GoaT CLI	0.2.5	https://github.com/genomehubs/goat-cli
Hifiasm	0.19.5-r587	https://github.com/chhylp123/hifiasm
HiGlass	44086069ee7d4d3f6f3f0012569789ec138f42b84 aa44357826c0b6753eb28de	https://github.com/higlass/higlass
Merqury.FK	d00d98157618f4e8d1a9190026b19b471055b22e	https://github.com/thegenemyers/MERQURY.FK
MitoHiFi	3	https://github.com/marcelauliano/MitoHiFi
MultiQC	1.14, 1.17, and 1.18	https://github.com/MultiQC/MultiQC
NCBI Datasets	15.12.0	https://github.com/ncbi/datasets
Nextflow	23.04.0-5857	https://github.com/nextflow-io/nextflow
PretextView	0.2	https://github.com/sanger-tol/PretextView
purge_dups	1.2.5	https://github.com/dfguan/purge_dups
samtools	1.16.1, 1.17, and 1.18	https://github.com/samtools/samtools
sanger-tol/ascc	-	https://github.com/sanger-tol/ascc
sanger-tol/genomenote	1.1.1	https://github.com/sanger-tol/genomenote
sanger-tol/readmapping	1.2.1	https://github.com/sanger-tol/readmapping
Seqtk	1.3	https://github.com/lh3/seqtk
Singularity	3.9.0	https://github.com/sylabs/singularity
TreeVal	1.0.0	https://github.com/sanger-tol/treeval
YaHS	1.2a.2	https://github.com/c-zhou/yahs

### Genome annotation

The Ensembl Genebuild annotation system ( Aken *et al.*, 2016) was used to generate annotation for the *Ceramica pisi* assembly (GCA_963859965.1) in Ensembl Rapid Release at the EBI. Annotation was created primarily through alignment of transcriptomic data to the genome, with gap filling via protein-to-genome alignments of a select set of proteins from UniProt ( UniProt Consortium, 2019).

### Wellcome Sanger Institute – Legal and Governance

The materials that have contributed to this genome note have been supplied by a Darwin Tree of Life Partner. The submission of materials by a Darwin Tree of Life Partner is subject to the **‘Darwin Tree of Life Project Sampling Code of Practice’**, which can be found in full on the Darwin Tree of Life website here. By agreeing with and signing up to the Sampling Code of Practice, the Darwin Tree of Life Partner agrees they will meet the legal and ethical requirements and standards set out within this document in respect of all samples acquired for, and supplied to, the Darwin Tree of Life Project.

Further, the Wellcome Sanger Institute employs a process whereby due diligence is carried out proportionate to the nature of the materials themselves, and the circumstances under which they have been/are to be collected and provided for use. The purpose of this is to address and mitigate any potential legal and/or ethical implications of receipt and use of the materials as part of the research project, and to ensure that in doing so we align with best practice wherever possible. The overarching areas of consideration are:

• Ethical review of provenance and sourcing of the material

• Legality of collection, transfer and use (national and international) 

Each transfer of samples is further undertaken according to a Research Collaboration Agreement or Material Transfer Agreement entered into by the Darwin Tree of Life Partner, Genome Research Limited (operating as the Wellcome Sanger Institute), and in some circumstances other Darwin Tree of Life collaborators.

## Data Availability

European Nucleotide Archive: *Ceramica pisi* (broom moth). Accession number PRJEB65408; https://identifiers.org/ena.embl/PRJEB65408 ( Wellcome Sanger Institute, 2023). The genome sequence is released openly for reuse. The *Ceramica pisi* genome sequencing initiative is part of the Darwin Tree of Life (DToL) project. All raw sequence data and the assembly have been deposited in INSDC databases. Raw data and assembly accession identifiers are reported in Table 1 and Table 2.

## Author information

Members of the University of Oxford and Wytham Woods Genome Acquisition Lab are listed here: https://doi.org/10.5281/zenodo.12157525.

Members of the Darwin Tree of Life Barcoding collective are listed here: https://doi.org/10.5281/zenodo.12158331

Members of the Wellcome Sanger Institute Tree of Life Management, Samples and Laboratory team are listed here: https://doi.org/10.5281/zenodo.12162482.

Members of Wellcome Sanger Institute Scientific Operations: Sequencing Operations are listed here: https://doi.org/10.5281/zenodo.12165051.

Members of the Wellcome Sanger Institute Tree of Life Core Informatics team are listed here: https://doi.org/10.5281/zenodo.12160324.

Members of the Tree of Life Core Informatics collective are listed here: https://doi.org/10.5281/zenodo.12205391.

Members of the Darwin Tree of Life Consortium are listed here: https://doi.org/10.5281/zenodo.4783558.
