# Supplementary material for: The genome sequence of the Broom moth, Ceramica pisi Linnaeus, 1758
Source: Wellcome Open Res. 2024 Sep 20;9:539. [Version 1] doi: 10.12688/wellcomeopenres.23050.1 (PMC11568375; doi:10.12688/wellcomeopenres.23050.1)
Supplement: Peer review history

# Reviewer report: Reviewer response for version 1

**DOI:** 10.21956/wellcomeopenres.25386.r106434

**Published:** 2024-11-15

**Authors:** Lino Ometto

**Affiliations:** 1 University of Pavia, Pavia, Italy

This manuscript by Griffits and colleagues provides a comprehensive description of the methods and results of a genome assembly from a male of Broom moth, *Ceramica pisi.*

 I only have two minor suggestions that the author may consider:

  

- Methods section: the sentence “Specimen ID SAN00002576 (ToLID ilCerPisi1) was used for PacBio HiFi sequencing and specimen ID SAN00002580 (ToLID ilCerPisi2)” seems truncated, I guess the second specimen was used for Hi-C sequencing (see Table 1), please specify.
- Page 8, Genome annotation: not clear what a “select set of proteins” corresponds to, please clarify.

Are sufficient details of methods and materials provided to allow replication by others?

Yes

Is the rationale for creating the dataset(s) clearly described?

Yes

Are the datasets clearly presented in a useable and accessible format?

Yes

Are the protocols appropriate and is the work technically sound?

Yes

Reviewer Expertise:

Inset genomics, population genetics

I confirm that I have read this submission and believe that I have an appropriate level of expertise to confirm that it is of an acceptable scientific standard.

# Reviewer report: Reviewer response for version 1

**DOI:** 10.21956/wellcomeopenres.25386.r106439

**Published:** 2024-10-21

**Authors:** Andrew J. Veale

**Affiliations:** 1 Manaaki Whenua Landcare Research, Lincoln, New Zealand

All aspects of the paper appear complete and appropriate.  I have no issues with the paper and feel it is ready to indexing.  The data appears excellent.  The only thing I can think to add, which is probably not necessary, is confirming that this species has a female ZW male ZZ chromosome system.

Are sufficient details of methods and materials provided to allow replication by others?

Yes

Is the rationale for creating the dataset(s) clearly described?

Yes

Are the datasets clearly presented in a useable and accessible format?

Yes

Are the protocols appropriate and is the work technically sound?

Yes

Reviewer Expertise:

wildlife ecology and genomics

I confirm that I have read this submission and believe that I have an appropriate level of expertise to confirm that it is of an acceptable scientific standard.
